# How changes in nutrition have influenced the development of allergic diseases in childhood

**DOI:** 10.1186/1824-7288-38-22

**Published:** 2012-05-31

**Authors:** Diego G Peroni, Beatrice Bonomo, Serena Casarotto, Attilio L Boner, Giorgio L Piacentini

**Affiliations:** 1 Pediatric Department, University of Verona, Ospedale G.B.Rossi, 37134, Verona, Italy

**Keywords:** Allergy, Children, Infant feeding, Diet, Nutrients, Anti-oxidants, Vitamin D

## Abstract

The increasing prevalence of allergic diseases in childhood in the last decades could be linked to concomitant dietary changes, especially with the modified and lower consumption of fruit, vegetables and minerals. The consumption of these foods by pregnant women and children in the first years of life seems to be associated with a reduced risk of asthma and related symptoms. Foods that can prevent the development of wheezing through their antioxidant effects contain vitamin C and selenium; blood levels of these elements correlate negatively with the risk of wheezing. Intake of vitamin E during pregnancy also appears to be correlated with a reduced risk of wheezing for the unborn child. Similarly, low intake of zinc and carotenoids by pregnant women is associated with an increased risk of wheezing and asthma in childhood. Fiber also has anti-inflammatory properties and protective effects against allergic diseases such as atopic dermatitis and asthma. The consumption of fat influences the development of the airways. Populations in Western countries have increased their consumption of n-6 PUFAs and, in parallel, reduced n-3 PUFAs. This has led to decreased production of PGE2, which is believed to have a protective effect against inflammation of the airways. Conflicting hypotheses also concern vitamin D; both an excess and a deficiency of vitamin D, in fact, have been associated with an increased risk of asthma. Further studies on the role of these substances are necessary before any conclusions can be drawn on a clinical level.

**Astratto:**

La crescente prevalenza negli ultimi decenni delle malattie allergiche in età pediatrica potrebbe essere legata a concomitanti cambiamenti nella dieta, in particolare alla minore e modificata introduzione di frutta, verdura e minerali. Il consumo di questi alimenti da parte delle donne in gravidanza e dei bambini nei primi anni di vita sembra essere associato ad un ridotto rischio di asma e di sintomi correlati. Gli alimenti che possono prevenire lo sviluppo di respiro sibilante (wheezing) attraverso i loro effetti antiossidanti contengono vitamina C e selenio; i livelli ematici di questi elementi sono correlati negativamente con il rischio di wheezing. Inoltre l'assunzione di vitamina E durante la gravidanza sembra essere correlato con un rischio ridotto di respiro sibilante per il nascituro. Allo stesso modo, basso apporto di zinco e di carotenoidi in donne in gravidanza è associata ad un aumentato rischio di wheezing e asma nell'infanzia. Anche le fibre hanno proprietà anti-infiammatorie ed effetti protettivi contro le malattie allergiche come la dermatite atopica e l’asma. Il consumo di grassi influenza lo sviluppo delle vie aeree. Le popolazioni dei paesi occidentali hanno aumentato il loro consumo di n-6 PUFA e, parallelamente, ridotto n-3 PUFA. Ciò ha portato alla diminuzione della produzione di PGE2, che si ritiene abbia un effetto protettivo contro l'infiammazione delle vie aeree. Ipotesi contrastanti riguardano la vitamina D, sia un eccesso che una carenza di vitamina D, infatti, sono stati associati ad un aumentato rischio di asma. Ulteriori studi sul ruolo di queste sostanze sono necessari prima di trarre conclusioni sul piano clinico.

## Introduction

The prevalence of allergic diseases in Western countries has shown a growing trend since the early 60s, with a growth rate ranging from 25% to 75% per decade [1-4], when it reached a plateau early in the new millennium [5]. This rapid increase in prevalence has stimulated the formulation of various hypotheses on the possible role that environmental factors play in this trend; changes in diet and the nutrient contents of foods have particularly been investigated. Asthma has been associated with a reduction of antioxidants in the diet (vitamin E, vitamin C, carotenoids, selenium, polyphenols and fruit, zinc), a reduction of n-3 PUFAs (polyunsaturated fatty acids) and an increase in n-6 PUFAs, and vitamin D supplementation or deficiency. There have been several observational and supplementation studies whose results were often contradictory and non-conclusive.

The modern diets of people living in industrialized countries have changed in terms of nutrient composition and food quality. Studies show that in Great Britain between 1967 and 2005 there was a decrease in the consumption of fruit, vegetables and saturated fats (butter), and an increase in the consumption of fruit juices, low-fat spreads and vegetable oils [6]. The nutrient contents were altered, with a decrease in the percentage of minerals from fruits and vegetables [7]. This may be due to the different modes of production, distribution and storage of foods than ever before. In the past, in fact, produce was grown and marketed locally and consumed soon after picking, but today longer periods of transport and storage are common and often necessary. It has been shown that vitamin C decreases by 65% in pineapple two weeks after harvest [8]. The flavonoid content of fruit grown in polyethylene tunnels is significantly reduced compared to that grown in the open air [6]. Nonetheless, most studies on fruits and vegetables indicate that their high consumption by pregnant mothers and children in the first years of life reduces the risk of developing asthma and asthma-related symptoms [9]. A woman's high adherence to the Mediterranean diet during pregnancy has a significant protective effect against the development of atopic wheezing (odds ratio = OR 0.3), persistent wheezing (0.22) and atopy (0.55), respectively, at the age of 6.5 years [10].

Recently, the interest of researchers has focused on the nutrition of pregnant women and children in the earliest years of life, on the assumption that diet may play a central role in the development of the airways and the immune system, possibly already in the womb, through epigenetic mechanisms [11-13]. Indeed, development of the lungs and airways is regulated by genetic and epigenetic factors that may be affected by the environment. For example, it has been documented that maternal smoking during pregnancy increases the risk of asthma in the unborn child [14]. These considerations support the hypothesis that the diet of pregnant women and children in the earliest years of life can influence, probably through epigenetic mechanisms, the epithelial and mesenchymal development of the lungs and airways [15,16]. In addition, asthma and allergic diseases are characterized by an imbalance of the immune response of T cells toward the T helper 2 (Th2) phenotype.

In this review we analyze various micronutrients and their possible association with atopic diseases.

## The role of folates

One of the most recent developments has been the recognition of the epigenetic effects of dietary folate on the development of asthma. Animal models demonstrated that folate supplementation during pregnancy induced hypermethylation (suppression) of regulatory genes in lung tissue, leading to the subsequent development of allergic airway disease [17]. In humans, one recent study reported that folate supplements during pregnancy were associated with an increased risk of wheezing and asthma in infants [18]; but another study found the opposite [19] to be the case. It is evident that there is an urgent call for additional studies regarding folates.

## The role of antioxidants

The observation that the increased prevalence of asthma has risen along with lower intake of antioxidants in the diet led Seaton et al. in 1994 to assume that the population had become more susceptible to asthma due to reduced antioxidant intake [20]. The proposed mechanisms are not only a reduced natural antioxidant defense capacity of the lungs [20], but also the ability of antioxidants to act on fetal development of the airways and/or on the first interaction between an allergen and the immune system. In mouse models, maternal supplementation of vitamin E accelerated the growth of the hypoplastic lung, increased lung complexity, air surface and gem number [21]. Zinc deficiency during pregnancy in rats was associated with abnormalities in the development of the lungs; or rather, the lungs were smaller, had reduced levels of DNA and a decreased calibre of alveolar ducts [22]. In addition, some metallo-proteinases (MMP-3, MMP-9, ADAM33), important for the airways, are zinc-dependent; so the intake of zinc during pregnancy could affect their activity [23].

Antioxidants may also affect the first interaction between antigens and the immune system [9]. Vitamin E appears to influence the activity of T cells through an indirect mechanism, increasing the activity of COX-2 and the production of prostaglandin E-2 (PGE-2) by macrophages, promoting the differentiation of T cells to Th-2 lymphocytes [9]. Vitamin E directly affects T cells by down-regulating the expression of interleukin-4 (IL-4) mRNA in T-cells [24]. Therefore, a deficiency of zinc in humans would be able to drive a differentiation of T lymphocytes in the Th-2 direction [25], whereas in animals it would increase the eosinophilia associated with inflammation of the allergic airways [26].

In 2001 Shaheen et al. [27] demonstrated in subjects aged 16 to 50 years a positive association between the intake of apples, red wine and asthma, indicating a probable protective effect of the flavonoides contained in apples and red wine. Flavonoids may reduce asthma inflammation through their antioxidant, anti-allergic and anti-inflammatory properties. Flavonoids are scavengers of nitric oxide [28] and can inhibit the release of histamine, arachidonic acid (AA) metabolism and production of cytokines [29]. The trans-resveratrol content in red wine can down-regulate the expression of the transcription factor NF-kB [30]. Moreover, kellin, a particular flavonoid, has bronchodilator properties [30]. A study by Okoko et al. in children between 5 and 10 years reported that the daily consumption of bananas and apple juice concentrate was associated with a reduced risk of wheezing but not of asthma [31].

Several observational studies have linked antioxidants with asthma and have investigated the possible effects of supplements on the treatment of asthma. However, the evidence that emerges from these studies is weak, due to possible bias and limitations, because cross-sectional and case–control studies predominate [32]. The limitations include the difficulties in quantifying the intake of nutrients in the diet, in measuring the concentrations of nutrients in the blood, the reverse causation (patients with asthma and allergic diseases can vary their diet) and other confounders [32,33]. Furthermore, studies that measured biomarkers of antioxidants in the blood are limited by the systemic oxidative stress associated with asthma, which reduces the concentration of these components in the blood [34].

Regarding vitamin C, a Cochrane review concluded that there is not enough evidence to recommend supplements in the treatment of asthma [35]. A more recent review and meta-analysis by Nurmatov et al. [36] confirmed that studies done on vitamin C and selenium so far do not support sufficient evidence for an association between intake of these nutrients and the prevalence of asthma in children. In particular, two large birth cohort studies, on 1290 [12] and 1924 mothers [37], respectively, showed that there was no association between wheezing in 2-year-old children and vitamin C intake during pregnancy, assessed through a semi-quantitative Food Frequency Questionnaire (FFQ) [38].

With regard to selenium, it was found that in Europe selenium intake with foods and consequently its blood levels has dropped; the cause is likely due to the increased consumption of European wheat, which is less rich in selenium compared to that of North America, as well as to changes in the technology of bread making [6].

Devereux, in a birth cohort study of 1924 mothers and their children, reported that high levels of selenium in maternal plasma at 12 weeks of gestation and in the newborns' umbilical cord blood were associated with a reduced risk of wheezing at 2 years of age, but not with asthma at 5 years [39]. The English Avon Longitudinal Study of Parents and Children (ALPSAC study) based on 2044 mothers and their offspring similarly concluded that high levels of selenium were associated with a reduced risk of persistent wheezing (OR 0.67) [40]. However, other four case–control studies [41-44] and two cross-sectional studies [45,46] were not able to demonstrate such significant associations. Randomized controlled trials on selenium [47] and vitamin E [48] supplements in adults with moderate to severe asthma reported no beneficial effect on the measured parameters. Positive results seem to regard vitamin E, for which a meta-analysis [36] of 3 major cohort studies [9,49,50] found a beneficial association between the intake of this nutrient by women during pregnancy and a reduction of the risk of wheezing at the age of 2 years (OR 0.68) [36].

Although zinc is not properly considered an antioxidant, Litonjua and Devereux in their cohort studies reported that low levels of zinc in the diet of women during pregnancy were associated with an increased likelihood that their children would develop wheezing and asthma in childhood [12,49].

## The role of fiber

Dietary fiber and oligosaccharides (acting as prebiotics) are important in promoting gut colonization. These non-digestible dietary components ferment to produce short-chain fatty acids (SCFAs) with anti-inflammatory properties [51]. Modern diets, which contain less fiber, thereby reducing substrates for the production of anti-inflammatory SCFAs, may be implicated in the failure of immune tolerance and in the propensity to develop both inflammatory autoimmune and allergic diseases [44]. Recent studies on prevention suggest a possible protective effect of administration of neonatal prebiotics for allergic outcomes such as atopic dermatitis, recurrent wheezing and allergic urticaria [52]. However, more studies are needed before recommendations can be made.

## The role of lipids

In 1997 Black and Shape observed that in Western countries the intake of saturated fats (butter and lard) had dropped in recent decades in favor of polyunsaturated fatty acids omega 6 (n-6 PUFA) contained in vegetable oils (sunflower seed, soy and corn oil) and margarine. This is probably a consequence of a coronary heart disease campaign by public health authorities [53]. At the same time, there was a lower intake of omega-3 polyunsaturated fatty acids contained in oily fish (anchovies, herrings, mackerels, sardines) and in cod liver oil [53]. Since this change in eating habits had occurred in tandem with the increased prevalence of asthma and allergic diseases, a link between the two phenomena has been proposed [53,54].

Several studies have assessed the effects of omega-6 and omega-3 in the diet on outcomes of atopic diseases. Some studies have focused on concentrations of polyunsaturated fatty acids in the breast milk of atopic mothers versus non-atopic mothers. These studies showed lower concentrations of AA in the breast milk of atopic mothers compared with healthy controls [55-63], although the difference was statistically significant in only 2 of these studies [58,60]. The low concentrations of AA were maintained for up to three months of lactation. A similar trend was shown for di-homo-gamma-linolenic acid (DGLA). Concentrations of long-chain fatty acids in the umbilical cord blood of infants born of atopic mothers showed no statistically significant results [64-67]. This type of studies is quite complex because the concentrations of PUFA in the blood can be affected not only by the maternal diet but also by the efficiency of placental transport and placental and fetal metabolism of PUFAs [68]. The ALSPAC study on 1238 infants detected a positive association between eczema, late-onset wheezing and the linoleic acid (n-6)/ alpha-linolenic acid (n-3) ratio [69]. However, due to the large number of comparisons made, the authors concluded that it is unlikely that fetal exposure to n-3 and n-6 PUFAs is so important in determining the onset of wheezing and atopic diseases. Yu et al. [64] found in their study that the development of atopic diseases and asthma during the first 6 years of life was not associated with the serum content of long-chain fatty acids in the blood of the umbilical cord.

Wijga et al [70] reported an increased prevalence of eczema (at 1 year), asthma (at 4 years) and persistence of symptoms of eczema and asthma in children of atopic mothers if the mother's milk contained low concentrations of alpha-linolenic acid (ALA), eicosapentaenoic acid (EPA), docosahexaenoic acid (DHA), total n-3 fatty acids or a low n-3/n-6 ratio. On the contrary, Oddy [71] and Laitienen [72] did not find any significant differences in the composition of n-3 and n-6 PUFAs, except for linoleic acid which was lower in the milk consumed by children that become atopic at 1 year [72].

Even if other factors, such as cytokines may be involved [73-76], dietary supplements of lipids in children with asthma have again produced conflicting results. Hodge, in a study of 39 children between 8 and 12 years old, found no improvement in the lung function of children supplemented with omega-3, but only a reduction in blood eosinophils and *in vivo* production of TNF-α by mononuclear cells [77]. Another study showed that in a group of children that took fish oil supplements there was a significant improvement in asthma scores and airway responsiveness compared to controls [78]. Other interesting results come from cohort studies also based on omega-3 supplementation. In the Childhood Asthma Prevention Study [79], 616 children at high risk of atopy were recruited after 6 months of life. Some were given omega-3 supplements, the others a placebo. The babies taking supplements showed a reduction in the incidence of wheezing at 18 months, wheezing for more than one week and visits to the doctor for wheezing. At the 5-year follow-up, the protective effects observed at 18 months had disappeared or were minimal [79]. It could be argued that supplementation in a 6-month-old child may be too late to influence the immune system. For this reason Dunstan et al. [80], in a randomized controlled trial, gave fish oil supplements to the pregnant mothers of 40 children at high risk of developing atopy. The children showed an overall reduction in the allergen-induced production of cytokines (IL-5, IL-10, IL-13, IFN-γ) by isolated cord blood mononuclear cells [80]. Concerning the clinical effects of supplementation, the authors only found reduced allergic sensitization to eggs in children at one year of age [80].

## The role of vitamin D

There are two conflicting hypotheses linking vitamin D to an increasing incidence of asthma and allergic diseases, according to the so-called "paradox of vitamin D". Both an excess (resulting from supplementation) and a deficiency (due to low solar exposure and the inability to compensate with diet) of vitamin D have been associated with an increased risk of asthma and allergies in Western countries [81]. Three birth cohort studies reported that low levels of vitamin D in the diet during pregnancy are associated with a higher risk of wheezing at the age of 16–24 months [82], 3 years [83] and 5 years [84], respectively. The authors of the last two studies [83,84], Weiss and coll., estimated that the population risk for asthma caused by a deficiency of vitamin D during pregnancy is around 40% [85]. The intake of vitamin D in these studies was assessed through a FFQ, but serum levels of 25-OH vitamin D were not measured [32]. Erkkola et al. reported that high levels of vitamin D in foods consumed by mothers while pregnant reduced the risk of asthma (OR 0.80) and allergic rhinitis (OR 0.85) in their off-spring at 5 years of age [86]. A recent report stated that cord blood 25-hydroxyvitamin D levels were inversely associated with the risk of respiratory infections and early childhood wheezing [87]. In contrast, Gale et al. reported that maternal exposure to 25-OH vitamin D concentrations above 75 mmol/L significantly increased the risk of asthma (OR 5.40) and atopy (OR 3.26) [88]. However, these results only referred to univariate models, with no adjustment for potential confounders; and there was also a significant loss of patients during the follow-up period (61.8%), especially at 9 years [89]. In agreement with these findings, Hyppönen study [90] found that high doses of vitamin D supplements (≥2000 IU per day) in the first year of life was associated with an increased risk of asthma (OR 1.35), allergic rhinitis (OR 1.66) and atopy (OR 1.46) later in life. However, this finding may be related to very high doses of vitamin D [91].

Further studies are necessary to clarify the role of vitamin D in the induction of immune tolerance and the subsequent risk of developing allergic diseases.

## Conclusions

From an epidemiological point of view allergic diseases and asthma have been strongly associated with modification of nutrients in the diet (vitamin E, vitamin C, carotenoids, selenium, polyphenols and fruit, zinc), a reduction of n-3 PUFAs (polyunsaturated fatty acids) and an increase in n-6 PUFAs, and vitamin D supplementation or deficiency. However, there are several observational studies whose results are often contradictory and non-conclusive. Data about the effects of supplementation with these nutrients during pregnancy or early life are particularly lacking. Therefore, we suggest that further studies on the role of these substances are necessary before any conclusions can be drawn on a clinical level.

## Abbreviations

PUFA, Polyunsaturated Fatty Acids; PGE-2, Prostaglandin E-2; AA, Arachidonic Acid; FFQ, Food Frequency Questionnaire; ALPSAC, Avon Longitudinal Study of Parents And Children; SCFAs, Short-Chain Fatty Acids; DGLA, Di-Homo-Gamma-Linolenic Acid; ALA, Alpha-Linolenic Acid; EPA, Eicosapentaenoic Acid; DHA, Docosahexaenoic Acid.

## Competing interests

The authors declare that they have no competing interests.

## Author’s contributions

DG Peroni and AL Boner designed the review and revised with GL Piacentini the final version of the manuscript. B Bonomo and S Casarotto generated the draft version of the paper. All authors approved the final version of the paper.
